# Salvage of a failed partial prosthetic breast reconstruction using a free deep inferior epigastric artery perforator (DIEP) flap

**DOI:** 10.1093/jscr/rjaf888

**Published:** 2025-11-12

**Authors:** Christopher Cherian, Sarah Omar, Krzysztof Sosnowski, Chloe Jordan, Charles Malata

**Affiliations:** Department of Surgery, C/O Princess Alexandra Hospital, Queen Elizabeth Avenue, The Quarter 2640, Anguilla, British West Indies; Department of Plastic and Reconstructive Surgery, Addenbrooke’s Hospital, Cambridge University Hospitals NHS Foundation Trust, Hills Rd, Cambridge CB2 0QQ, United Kingdom; Department of Plastic and Reconstructive Surgery, Addenbrooke’s Hospital, Cambridge University Hospitals NHS Foundation Trust, Hills Rd, Cambridge CB2 0QQ, United Kingdom; Cambridge Breast Unit, Addenbrooke’s Hospital, Cambridge University Hospitals NHS Foundation Trust, Hills Rd, Cambridge CB2 0QQ, United Kingdom; School of Clinical Medicine, University of Cambridge, Hills Rd, Cambridge CB20SP, United Kingdom; Department of Plastic and Reconstructive Surgery, Addenbrooke’s Hospital, Cambridge University Hospitals NHS Foundation Trust, Hills Rd, Cambridge CB2 0QQ, United Kingdom; Cambridge Breast Unit, Addenbrooke’s Hospital, Cambridge University Hospitals NHS Foundation Trust, Hills Rd, Cambridge CB2 0QQ, United Kingdom; Department of Plastic and Reconstructive Surgery, Addenbrooke’s Hospital, Cambridge University Hospitals NHS Foundation Trust, Hills Rd, Cambridge CB2 0QQ, United Kingdom; Cambridge Breast Unit, Addenbrooke’s Hospital, Cambridge University Hospitals NHS Foundation Trust, Hills Rd, Cambridge CB2 0QQ, United Kingdom; Anglia Ruskin University School of Medicine, Anglia Ruskin University, East Rd, Cambridge CB1 1PT, United Kingdom

**Keywords:** partial breast reconstruction, tertiary (salvage) breast reconstruction, prosthetic breast reconstruction, DIEP flap, severe capsular contracture, radiotherapy

## Abstract

Tertiary breast reconstruction with free tissue transfers is the gold standard in managing recalcitrant complications of implant-based whole breast reconstructions (WBRs). Whilst the salvage of implant-based WBR with free tissue transfers is well established, there have hitherto been no reports of free flap use for salvaging failed or suboptimal partial prosthetic breast reconstructions. We report a case of delayed partial breast reconstruction after lumpectomy and axillary clearance, where a deep inferior epigastric artery perforator flap successfully salvaged a suboptimal thoracodorsal artery perforator flap (TDAP) with underlying peri-implant capsular contracture, avoiding the need for completion mastectomy. The challenges encountered were complicated by severe capsular contracture, chronic pain, poor cosmesis, prior tissue rearrangement, radiotherapy changes, residual breast tissue, and scarring from a previous TDAP flap. Microvascular autologous conversion, a standard treatment of failed prosthetic WBRs, also has the versatility to salvage complex partial reconstructive dilemmas accentuated by prior radiotherapy and the presence of residual potentially compromised breast tissue.

## Introduction

Breast reconstruction following cancer ablation can either be partial (following lumpectomies) or total (following mastectomy). Total reconstruction is performed using prostheses, the patient’s tissues, or a combination of these modalities. Partial breast reconstruction mainly uses local tissues such as parenchymal rearrangement, therapeutic mammaplasty, or local flaps, with prostheses used occasionally [1–3].

With respect to tumour ablation, partial breast reconstruction can be immediate or delayed [4, 5]. When performed with implants, partial reconstruction carries risks from utilizing a foreign body, and its implantation in a previously, or soon-to-be, irradiated field [6]. These include infection, exposure, migration, capsular contracture (CC), and rupture [7]. Salvage surgery after failed implant-based breast reconstruction presents a unique series of challenges, including post-radiotherapy scarring, the presence of residual breast tissue, and prior axillary clearance (ANC) [8]. It can also be compounded by previous use of local flaps, which might have compromised tissue perfusion or caused considerable scarring. Similarly, the LD flap vascular pedicle might have been damaged during ANC or local flap dissection [9].

As a last resort, salvage can be undertaken by total autologous conversion, akin to failed or suboptimal post-mastectomy implant reconstructions [8, 10, 11]. This report highlights a case of a partial prosthetic breast reconstruction complicated by severe deformity and symptomatic CC and prior TDAP flap, which was salvaged with an free tissue transfer (FTT).

## Case report

**Figure 1:**
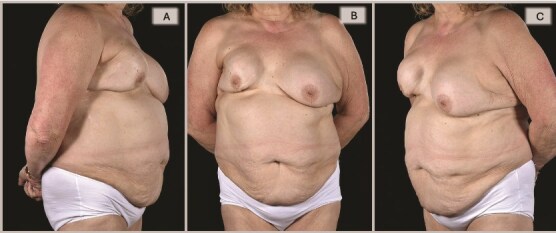
Preoperative evaluation for DIEP flap salvage revealed a contracted, high-riding right breast with a significantly narrowed footprint, lateral midaxillary scar with contour deformity, marked asymmetry in size, shape, and positions of the inframammary folds and nipple-areolar complexes, and severe capsular contracture, while the contralateral left breast also exhibited significant capsular contracture, in a patient with multiple comorbidities including asthma with barrel-shaped trunk, obesity (BMI 31), heavy smoking, type 2 diabetes, atrial fibrillation, and scoliosis.

**Figure 2:**
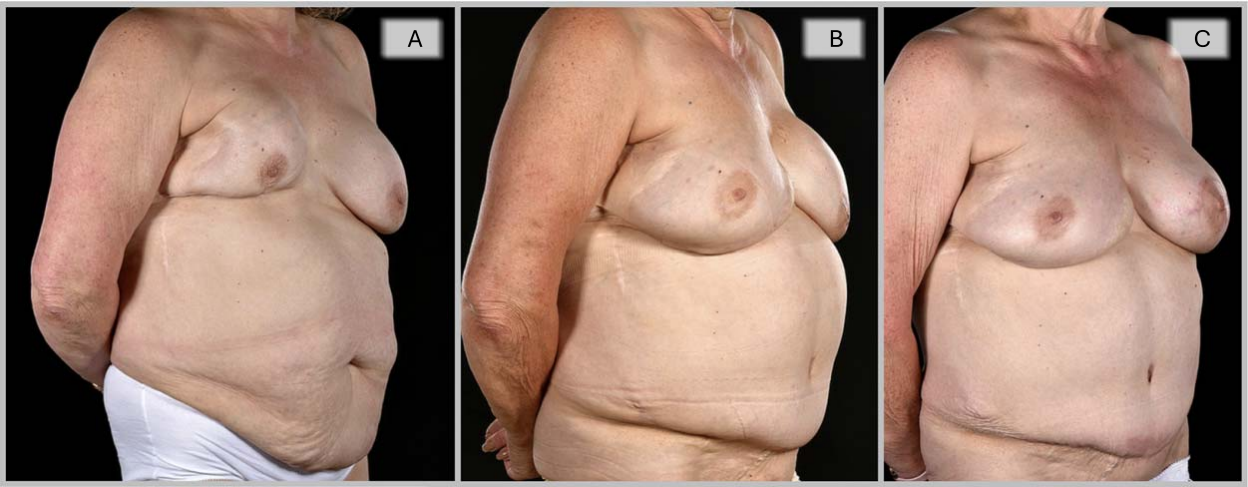
Right oblique views showing the pre-, 6-, and 15-month post-operative appearances: (A) Severely contracted and scarred right breast prior to DIEP reconstruction, (B) 6 months post-DIEP: the flap has provided adequate volume and better IMF definition with dramatic transformation of its appearance, and (C) the symmetry was further improved by a contralateral balancing batwing mastopexy and implant exchange, with notable improvement in overall symmetry (in terms of NAC position, natural-looking upper poles, and overall volume).

**Figure 3:**
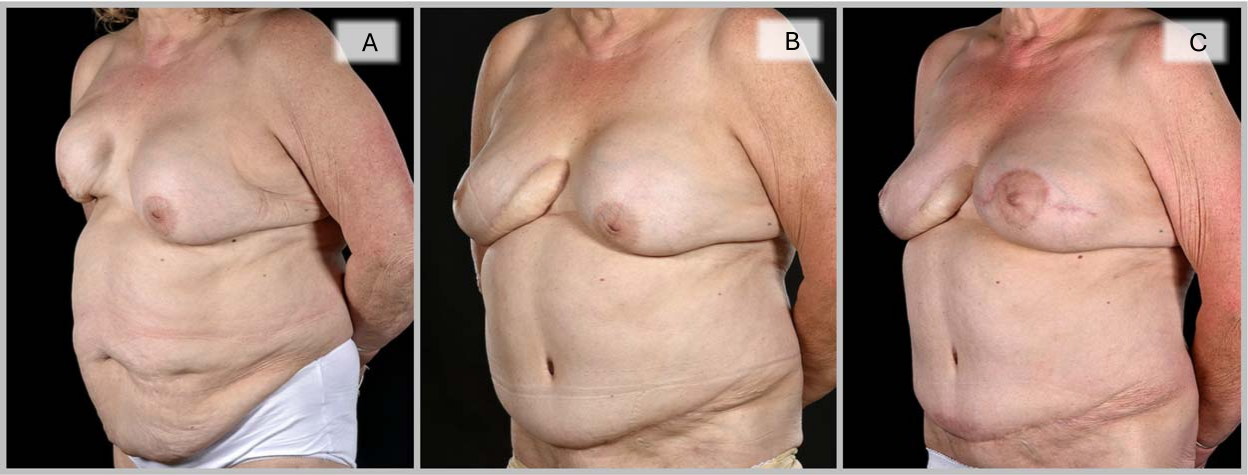
Left oblique views demonstrate the improvement achieved by DIEP reconstruction in correcting the severely deformed, high-riding, and contracted right breast with inferior skin shortage, restoring both volume and skin envelope for improved breast shape, with the inferomedial flap monitoring buoy skin paddle became less noticeable with time (3C) and a subsequent bat-wing mastopexy enabled the alignment of the two NACs (3C).

A 65-year-old woman was referred to the plastic surgery unit of a tertiary hospital for severe right breast pain and dissatisfaction with breast appearance (Fig. 1). At age 18, she had left breast implant augmentation for correction of developmental breast asymmetry. Twenty-five years later, she was diagnosed with invasive right breast cancer and underwent a lumpectomy with implant insertion and ANC, followed by chemotherapy and radiotherapy. Eight years later, she became unhappy with her right breast cosmesis and sought corrective surgery during which the implant was replaced along with a TDAP flap. Twelve years later, she presented with a persistent painful deformity of her right reconstructed breast and a contracture of the opposite breast. (Figs 1A–C, 2A, and  3A).

**Figure 4:**
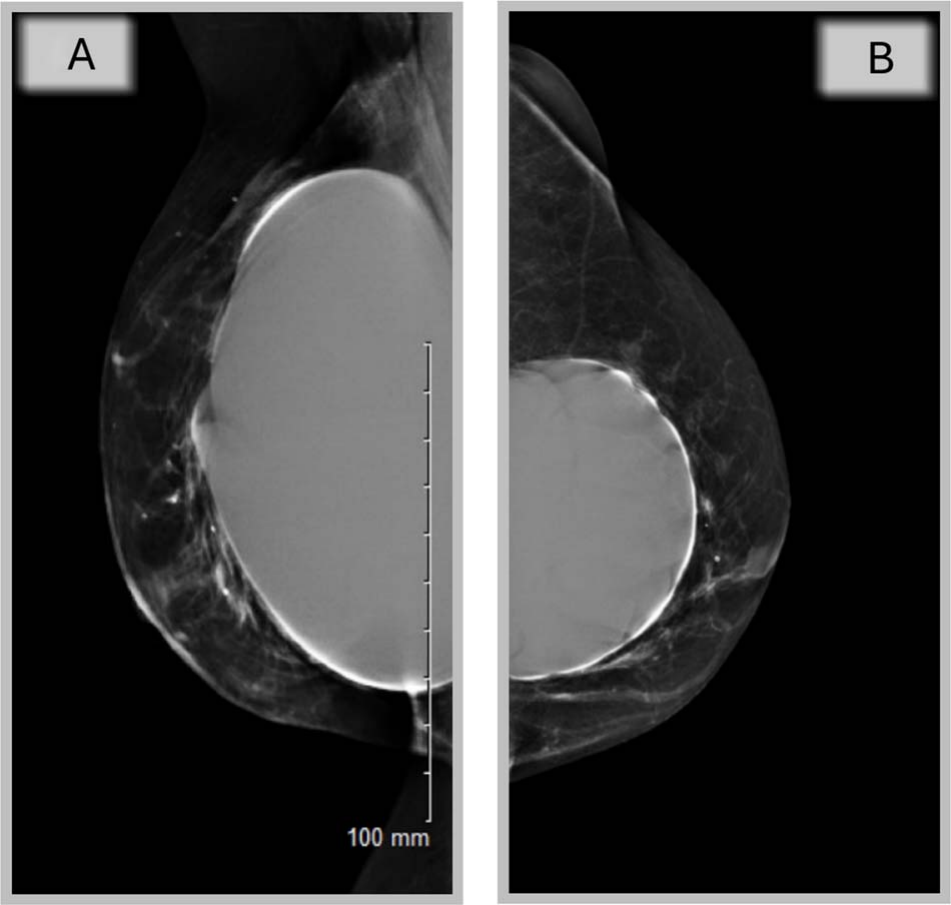
Mammography of the two breasts shows asymmetrical augmentation of the two breasts with no evidence of implant rupture or parenchymal abnormality supporting oncological MDT’s recommendation.

A completion mastectomy was recommended to facilitate whole breast reconstruction (WBR), but oncological multidisciplinary team (MDT) revealed no indication (Fig. 4A and B).

**Figure 5:**
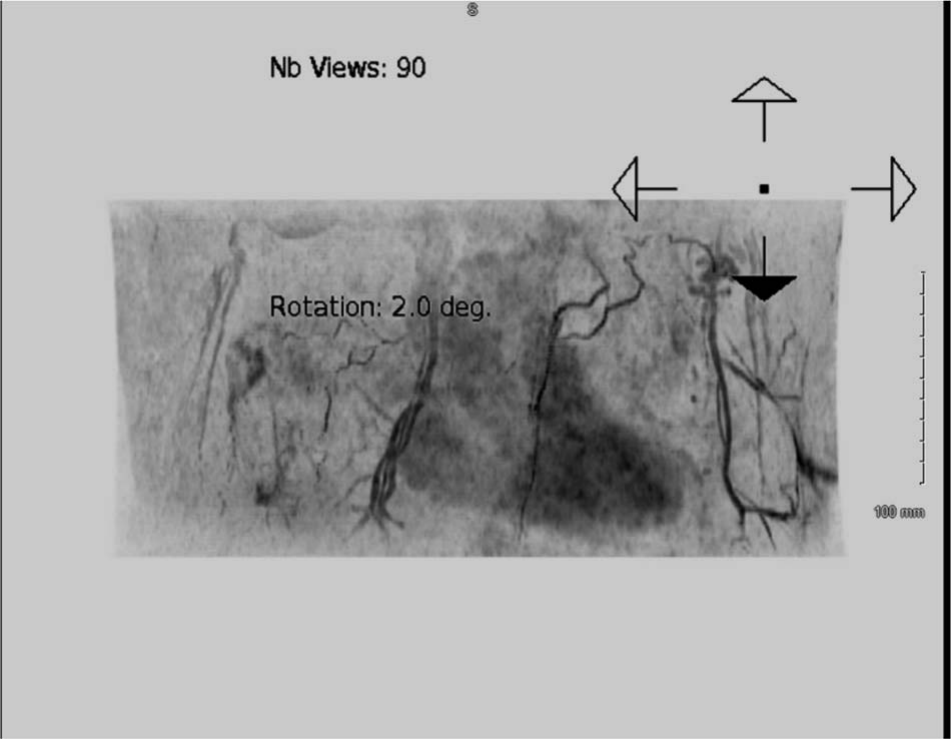
Pre-operative computed tomography (CT) angiography demonstrated robust vasculature perfusing the superior and lateral margins of the right breast, suggesting the planned infero-medial access incision would not de-vascularize the remaining breast tissue.

**Figure 6:**
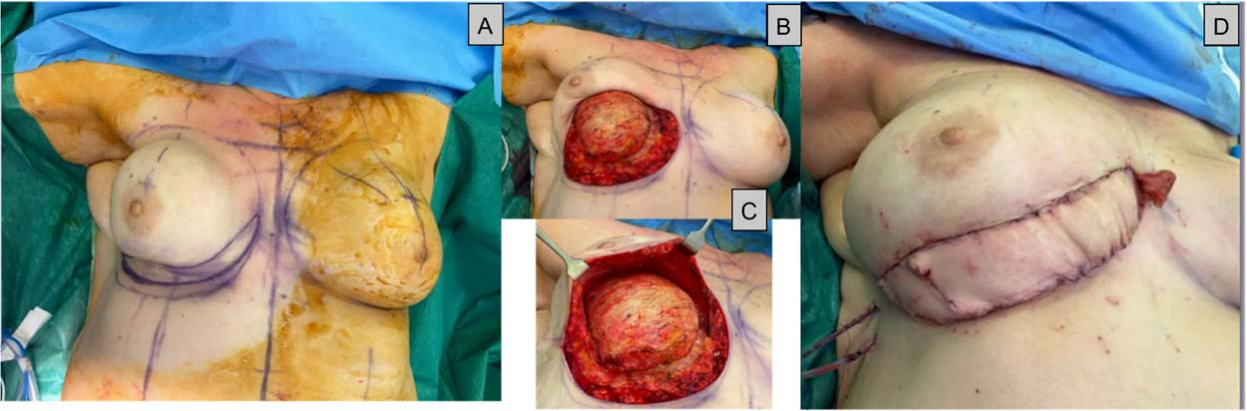
(A) Intraoperative series during DIEP surgery, a: Showing the inferior ellipse of contracted skin to be excised, (B + C) capsule undergoing total capsulectomy with the superior breast tissue still intact (D): Immediately after flap inset showing the monitoring skin paddle.

**Figure 7:**
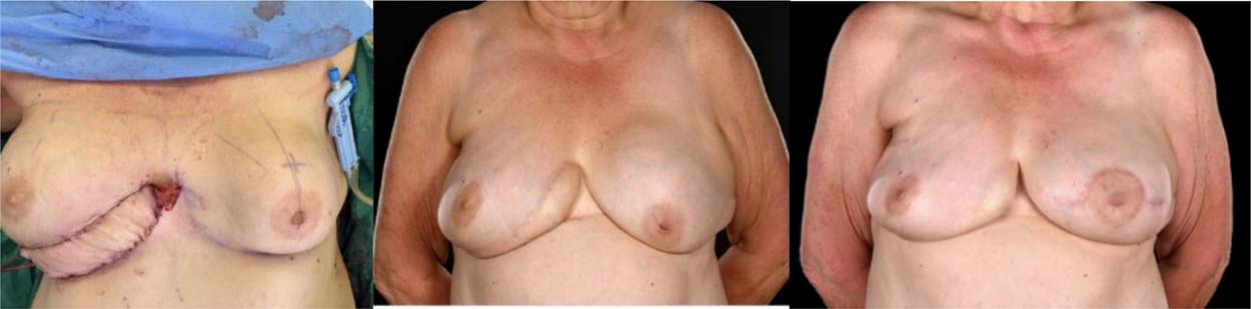
The intraoperative the 6- and 15-month post-operative photos demonstrate the evolution in the appearance of the monitoring paddle, which is finally well incorporated into the contouring of the breast, with most of the DIEP flap deeply buried in the capsulectomy pocket.

Given her previous loco-regional operations, the deep inferior epigastric artery perforator (DIEP) flap was deemed the most reliable source of skin and volume. An infero-medial surgical access was believed to be the safest approach (Fig. 5) and would enable a reconstruction with a right-sided DIEP flap, buoy skin paddle (Figs 6 and 7), following scar excision, capsulectomy, and explantation (Fig. 6). End-to-end anastomoses with the internal mammary vessels in the 2nd space were achieved, along with augmenting the venous drainage with a retrograde superior inferior epigastric vein-internal mammary vein (SIEV-IMV) anastomosis. Her recovery was complicated by basal atelectasis and pneumonia, causing post-operative delirium, before being discharged on the 8th postoperative day.

By 6 months, she was pain-free with a soft right breast and pleased with the cosmetic outcome (Figs 1–3). Fifteen months post-reconstruction, a contralateral symmetrizing capsulectomy, implant exchange, and simultaneous bat-wing mastopexy were performed (Fig. 7). Both of her breasts remain contracture-free free soft, and pain-free. (Figs 1–3,).

## Discussion

Total autologous conversion of failed, symptomatic, or suboptimal implant-based WBRs is a well-established concept [8, 10–13]. Often, such patients would have undergone several revisions before agreeing to the removal of the troublesome implant and replacement with an FTT [10–12, 14]. Currently, autologous tissue remains the first option in primary reconstruction of partial breast defects [3–5]. Our literature review identified reports of implant-based WBR salvaged with autologous tissue, but none describing autologous tertiary reconstruction of partial breast defects after implant removal [8]. This case documents a novel approach to this using an FFT to replace a capsulectomy-explantation defect and simultaneously augment native tissue.

Partial breast reconstructions are ideally performed at the time of lumpectomy before radiotherapy [4–6]. Implant-based partial breast reconstruction after lumpectomy, as in this patient, is rare due to the usually small defects and the absence of custom implants [1, 2]. Our patient received radiotherapy with the implant in place, developed complications underwent TDAP flap revision and implant replacement, but recalcitrant pain and intractable recurrent CC necessitated salvage DIEP reconstruction. Completion mastectomy with immediate flap reconstruction would have been an easier alternative, but there was no oncological indication [15].

Local flaps were considered for volume replacement, but previous TDAP use and lack of a reliable LD flap precluded their selection. The LTAP flap could not be used as its supply could have been damaged during the ANC and TDAP flap harvest. Of the potential donor sites assessed, the DIEP flap was chosen as the most feasible option due to sufficient tissue volume, robust blood supply, and available skin.

Partial breast reconstruction with a DIEP flap following previous breast-conserving surgery and radiotherapy is challenging. Flap inset must balance microsurgical access with preservation of native tissue vascularity. To reduce the risk of devascularization of the remaining breast tissue, the inferomedial incision was skewed superiorly for recipient vessel access, leaving a wide perfusing base. The lateral approach was avoided because dense, relatively avascular scar tissue made the thoracodorsal vessels unsuitable as recipients. Similarly, a central inframammary crease access incision was not utilized, as it would have made access to the recipient vessels more difficult. It was managed using a ‘Buoy Flap’ placed at the inferomedial incision, though implantable Dopplers are an alternative. Another difficulty was vessel exposure and performance of the microanastomoses due to silicone lymphadenitis, together with associated scarring of the chest wall from prior extensive tissue manipulation and radiotherapy.

Salvage reconstruction of failed prosthetic breasts is increasingly common as implant-based reconstructions rise worldwide. However, free flap partial reconstruction salvage in the context of radiotherapy and implant complications is unique and should be considered an option when faced with such challenges.

This approach can be a definitive, oncologically appropriate and safe solution for carefully selected, evaluated and counselled patients, who it is recommended should be referred to tertiary centres, not least because, by virtue of their complicated medical and surgical histories, are intolerant of multiple attempts at implant revisions, whilst free flap salvage provides a permanent solution.
